# A novel encryption scheme for high-contrast image data in the Fresnelet domain

**DOI:** 10.1371/journal.pone.0194343

**Published:** 2018-04-02

**Authors:** Nargis Bibi, Shabieh Farwa, Nazeer Muhammad, Adnan Jahngir, Muhammad Usman

**Affiliations:** 1 Department of Mathematics, COMSATS Institute of Information Technology, Wah Cantt., Pakistan; 2 Department of Computer Science, Fatima Jinnah Women University, Rawalpindi, Pakistan; 3 Department of Engineering Sciences, Ghulam Ishaq Khan Institute of Engineering Sciences and Technology, Sawabi, 23640, Pakistan; Lanzhou University of Technology, CHINA

## Abstract

In this paper, a unique and more distinctive encryption algorithm is proposed. This is based on the complexity of highly nonlinear S box in Flesnelet domain. The nonlinear pattern is transformed further to enhance the confusion in the dummy data using Fresnelet technique. The security level of the encrypted image boosts using the algebra of Galois field in Fresnelet domain. At first level, the Fresnelet transform is used to propagate the given information with desired wavelength at specified distance. It decomposes given secret data into four complex subbands. These complex sub-bands are separated into two components of real subband data and imaginary subband data. At second level, the net subband data, produced at the first level, is deteriorated to non-linear diffused pattern using the unique S-box defined on the Galois field $F_{2}^{8}$. In the diffusion process, the permuted image is substituted via dynamic algebraic S-box substitution. We prove through various analysis techniques that the proposed scheme enhances the cipher security level, extensively.

## Introduction

Data encryption is an integral part of multimedia technologies. Usually, internet users require to deliver, obtain, or store secret information [1]. Extensive information exchange over internet demands safety from unwanted surveillance, thieving, and bogus publicity [2]. In this frame of reference, images are exposed to ciphers which are unrecognizable by the human eye [3]. The highest familiar way to escape from any trouble is to convert the confidential data into dummy formation [4]. The information appeared can only be restored by those who know the reverse scheme used to attain the initial pattern with exclusive key specification [5–13]. Data encryption enables data integrity, confidentiality, and data authentication [14]. This is achieved using a digital data encryption which transforms the meaningful information into dummy data. This strategy has been evolved to preserve the information and is heavily based on cryptography [4].

Cryptography is used for data confidentiality. Claud Shannon [15], introduced the *substitution*- *permutation network* (SPN), each layer of which uses substitution followed by permutation. Cryptographic procedures are helpful to preserve the secret information by scrambling it into an uncorrelated data [4]. Improved security and confidentiality can be served if the two schemes, confusion and diffusion, are joined together into one hybrid system [16–18]. The gradual development of cryptographic systems witnesses the worth of the substitution box (*S* − *box*). It plays the role of a standout in symmetric key cryptography and a predominant source to produce nonlinearity [1, 19]. The indispensable involvement of the S-box demands new construction algorithms and the recent literature introduces some safer and more reliable S-boxes [1, 2, 20–24]. Further applications of S boxes in digital image ciphering, water marking as well as in steganography have become quite popular as well [3, 25–30]. In this work, we propose an efficient strategy that uses the algebraic properties of the Galois field to structure an S-box which exhibits extra-ordinary features as compared to some prevailing designs at a very low computational labour.

In recent literature, many innovative and advanced techniques of image encryption have become pivot of attention. Liu and Wang introduced image encryption algorithms based on one-time keys [31] and spatial bit-level permutations along with chaotic systems [32]. In [33], a chaotic encryption scheme based on perceptron model is proposed. [34] describes an encryption strategy using DNA complementary rule with chaos. Some of the most recent image encryption techniques are detailed in [35–42]. However, according to our learning there is no previous work on the applications of the Fresnelet transform in conjunction with S-box cryptography. In this paper, we use the cryptography to manipulate the significant information of the secret data.

Later part of this paper is ordered as follows. Section Fresnelet Transform provides a theoretical explanation of the Fresnelet transform. Section Algorithm for algebraic S-box describes the detailed algorithm used to develop an algebraic S-box. The cryptographic forte of the S-box is examined in Performance analysis of S-box, through some highly significant parameters. The remaining study present a comprehensive model for encryption and decryption, including simulations and evaluations. Lastly, Conclusion Section concludes the paper.

## Fresnelet transform

The propagation phenomena of wave are structured via diffraction principle using the Fresnel transform [43]. The Fresnel transform in wavelet domain produces the bases of Fresnelet transform. These bases are used to reconstruct the digital off axis hologram with certain composition of parameters. The parameters can be tuned to desired value of the resolution scale, adjusted with particular wavelength and the specific distance between observing plane to the propagating objects. Fresnelet transform is demonstrated to simulate the approximation the monochromatic waves propagation. Propagation of monochromatic waves is shown as a function $\Lambda \in \Omega_{2}(R)$, with the Fresnel transform model in terms of convolution integral as follows:

$$\begin{array}{c} {\tilde{\;\Lambda }}_{\tau }(p)=(\;\Lambda *k_{\tau })(p)\;\text{with}\;k_{\tau }(p)=\frac{1}{\tau }exp(i\pi \frac{p^{2}}{\tau^{2}}), \end{array}$$ (1)

The kernel *k*_*τ*_(*p*) is the one-dimensional prorogation model with given parameter *τ* > 0 in (2), where, *τ* depends on the propagating distance *d*, based on the wavelength value λ as shown:

$$\begin{array}{c} \tau =\sqrt{\lambda d}. \end{array}$$ (2)

Moreover, the kernel *k*_*τ*_(*p*) can be extended to two-dimensional wave propagation using the tensor product for $\Lambda \in \Omega_{2}\left(R^{2}\right)$, as follows:

$$\begin{array}{c} {\tilde{\Lambda }}_{\tau }(p,q)=(\Lambda *K_{\tau })(p,y), \end{array}$$ (3)

$$\begin{array}{c} K_{\tau }(p,q)=k_{\tau }(p)k_{\tau }(q). \end{array}$$ (4)

The *K*_*τ*_(*p*, *q*) is also known as the separable kernel to cover the two-dimensional Fresnel transform’s family [14]. To obtain the precise reconstruction of decomposed data, the Fresnel transform unitary property facilitates, prominently. Furthermore, the extension of the separable nature of one-dimensional wavelet transform into two-dimensional wavelet transform can be achieved using the Riesz basis [43]. The Riesz basis for $\Omega_{2}(R)$ of a two-parameter ${\left\{\psi_{j,l}\right\}}_{j,\;l\in \mathbb{Z}}$ can be defined on $\Omega_{2}(R)$ as family of the wavelet transform can be defined in terms of convolution integrals, such that

$$\begin{array}{c} {\left\{\psi_{j,l}\left(p\right)=2^{j/2}\psi \left(2^{j}p-l\right)\right\}}_{j,\;l\in \mathbb{Z}}. \end{array}$$ (5)

where, the Haar wavelet is used as an orthonormal basis generation for $\Omega_{2}(R)$. This is the simplest form of a multi-resolution decomposition by the wavelet transform, and can be used to get the desired reconstruction of the input original data as well [44]. This composition leads to the Fresnelet basis using the the Haar wavelet in combination of the Fresnel transform as follows:

$$\begin{array}{c} {\left\{{\left(\psi_{j,l}\right)}_{\tau }^{∼}\right\}}_{j,\;l\in \mathbb{Z}} \end{array}$$ (6)

$$\begin{array}{c} {\left(\psi_{j,l}\right)}_{\tau }^{∼}(p)=2^{j/2}{\tilde{\psi }}_{2^{j}\tau }(2^{j}p-l).\;\; \end{array}$$ (7)

For constant value of *τ*, an orthonormal basis in terms of Fresnelet transform can be obtained using: $\beta_{j,l}(p)={\left(\psi_{j,l}\right)}_{\tau }^{∼}(p)$, as follows:

$$\begin{array}{c} \Lambda =\sum_{j,l}\;\nu_{j,l}\;\beta_{j,l} \end{array}$$ (8)

$$\begin{array}{c} \;\;\nu_{j,l}=\langle \Lambda ,\beta_{j,l}\rangle . \end{array}$$ (9)

The coefficients representation using Fresnelet is done by *ν*_*j*, *l*_ in (9). Moreover, the separable nature is extended the Fresnelet transform’s from one-dimensional data to two-dimensional data. Following this, the four combinations are obtained using the tensor product $\mu_{\tau }^{\left(ll\right)},\;\mu_{\tau }^{\left(lh\right)},\;\mu_{\tau }^{\left(hl\right)},\;\text{and}\;\mu_{\tau }^{\left(hh\right)},$ for producing the approximation (lower-lower subband) and three high frequency details (lower-high subband, high-lower subband, and high-high subband) as follow:

$$\begin{array}{c} \mu_{\tau }^{\left(ll\right)}={\left(x_{j,l}\right)}_{\tau }^{∼}\left(p\right){\left(x_{j,l}\right)}_{\tau }^{∼}\left(q\right), \end{array}$$ (10)

$$\begin{array}{c} \mu_{\tau }^{\left(lh\right)}={\left(x_{j,l}\right)}_{\tau }^{∼}\left(p\right){\left(y_{j,l}\right)}_{\tau }^{∼}\left(q\right), \end{array}$$ (11)

$$\begin{array}{c} \mu_{\tau }^{\left(hl\right)}={\left(y_{j,l}\right)}_{\tau }^{∼}\left(p\right){\left(x_{j,l}\right)}_{\tau }^{∼}\left(q\right), \end{array}$$ (12)

$$\begin{array}{c} \mu_{\tau }^{\left(hh\right)}={\left(y_{j,l}\right)}_{\tau }^{∼}\left(p\right){\left(y_{j,l}\right)}_{\tau }^{∼}\left(q\right). \end{array}$$ (13)

In (10)–(13), the representation of scaling functions is done using *x* and representation of the wavelet functions is done using *y*. These functions are established a low-low filter in (10) and high details filter in (11)–(13). Following this, the four Fresnelet coefficients are generated using the basis function *μ* to data Λ, as follows:

$$\begin{array}{c} \begin{array}{cc} \Lambda_{\tau ,d}^{\left(ll\right)}=\langle \Lambda ,\mu_{\tau }^{\left(ll\right)}\rangle , & \Lambda_{\tau ,d}^{\left(lh\right)}=\langle \Lambda ,\;\mu_{\tau }^{\left(lh\right)}\rangle , \end{array} \end{array}$$

$$\begin{array}{cc} \Lambda_{\tau ,d}^{\left(hl\right)}=\langle \Lambda ,\mu_{\tau }^{\left(hl\right)}\rangle , & \Lambda_{\tau ,d}^{\left(hh\right)}=\langle \Lambda ,\mu_{\tau }^{\left(hh\right)}\rangle . \end{array}$$

The low-low filter data is demonstrated using the coefficient measure $\Lambda_{\tau ,d}^{\left(ll\right)}$ and the high-details data are represented using the coefficients measure $\Lambda_{\tau ,d}^{\left(lh\right)}$, $\Lambda_{\tau ,d}^{\left(hl\right)}$, and $\;\Lambda_{\tau ,d}^{\left(hh\right)}$, respectively. Theses Fresnelet coefficients are used to propagate the input image data. It transforms the input image data from meaningful information to dummy image using the Fresnelet forward transform in the form four complex subbands data [14].

**Fig 1:**
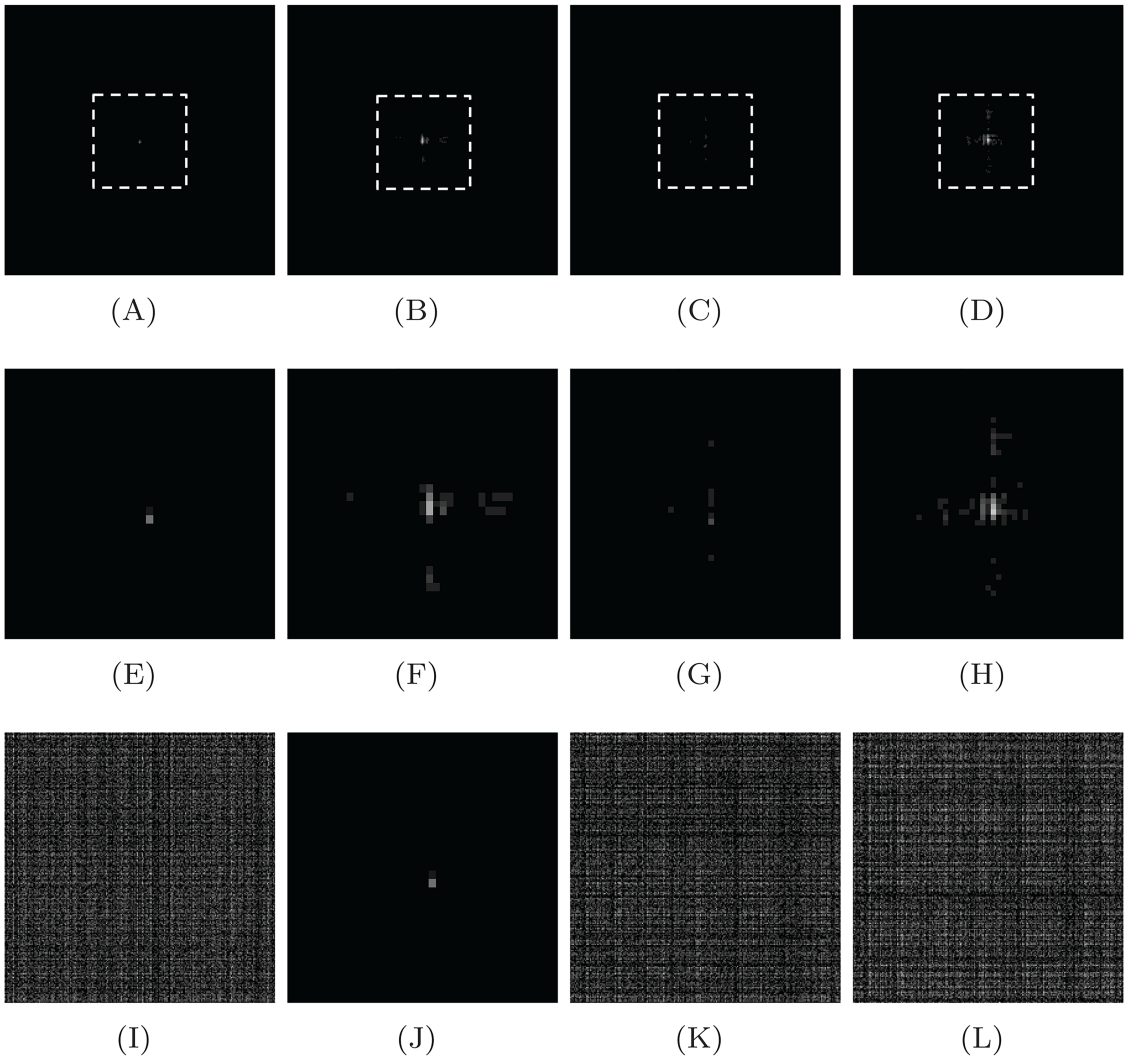
Fresnelet transform encryption of the given data USAF where the the magnitude of it is obtained using the Fresnelet coefficients with key parameter *d*_1_ = 1*m* as follows. (first row) approximation, horizontal data, vertical data, and diagonal data. (Second row) shows the zooming vision of the corresponding images listed in the first row. (Last row) represents the inverse magnitude using Fresnelet transformed data to the four subbands listed in the first row using key *d*_2_ = .01*cm*.

**Fig 2:**
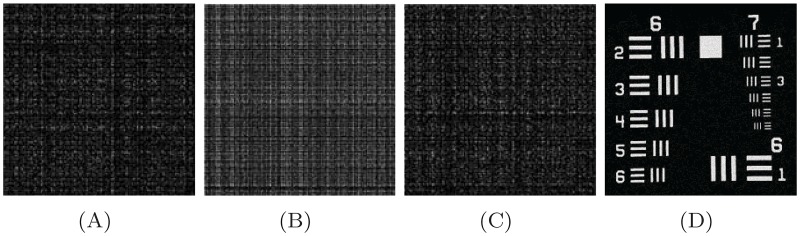
The scrambled information in last row of Fig 1 is reconstructed by combining all subband data is demonstrated in (A). It’s pixel value is composed in terms of the magnitude with complex valued data. The (B) and (C) are the real and imaginary parts of the dummy data displayed in (A). (D) The reconstructed image usaf is obtained using Fresnelet transform application with same parameter keys as in encryption case.

The Fresnelet transform unitary property is used in reconstruction of secret data by applying the conjugate transpose to encrypted data as shown in Fig 1. This outcome has a complex form of decrypted data. The decomposition stage of secret data of USAF image is shown in first row of Fig 2 as a 4 subbands based on the Forward Fresnelet transform with distance *d*_1_. The marked area in first row is demonstrated in second row as a zoomed-in region. This show the diffusion of meaningful data into meaning less dummy data. The inverse propagation of the first row of Fig 1 is propagated at distance *d*_2_. This inverse processing is termed as inverse Fresnelet transform. The four subbands in Fig 1(i)–1(l) are merged into complex data formation as a single image and its magnitude is shown in first image of Fig 2. The communication of encrypted data in digital form is distributed into real parts only. Therefore, the complex data is partition into two parts: real part *a* and magnitude of imaginary part *b* (in second and third image of Fig 2). Moreover, on reconstruction stage the imaginary part *b* (magnitude value) with *i* is added up into *a* (real value) to obtain the complex data using inverse process.

To transfer the given data with huge isolation and upgraded discreetness, the presented method implies the Fresnelet transform that holds distance parameters and the wavelength as keys, that are necessary for reconstruction of the correct information. Furthermore, the initial pattern of the information data is obtained in the reconstruction stage with the accurate parameters use during the inverse Fresnelet procedures.

## Algorithm for algebraic S-box

In this section, we describe the algorithm used for construction of our S-box. Generally, S-box is considered as the most influential component to produce the nonlinearity effect that relates to the confusion creating capability in ciphering. In order to grasp the structural properties of an S-box, we first need to go through some fundamental facts.

An *n* × *n* S-box can be defined as a vector Boolean function $F:F_{2}^{n}\to F_{2}^{n}$.

The algebraic structure of the background Galois field used to construct an S-box, plays very basic role. For the underlying construction of 8 × 8 S-box, we use $F_{2}^{8}=F_{2}\left[X\right]/<p\left(X\right)>$ with $p\left(X\right)=X^{8}+X^{6}+X^{5}+X^{4}+1\in F_{2}\left[X\right]$ is a degree 8 irreducible polynomial. It is worth mentioning that in advanced encryption standard, AES S-box algorithm uses $F_{2}^{8}$ based on *X*^8^ + *X*^4^ + *X*^3^ + *X* + 1. In fact, any degree 8 irreducible generating polynomial could be used for the field $F_{2}^{8}$ but this choice obviously affects the calculations.

We define the general linear group GL(n,F) as a group formed by all *n* × *n* invertible matrices over the field F. A projective general linear group of degree *n* over a field F is defined to be the quotient of GL(n,F) by its center. For this paper, we form the 8 × 8 S-box by considering the action of the aforementioned Galois field $F_{2}^{8}$ on $PGL(2,F_{2}^{8})$, i.e. we take a function $f:PGL\left(2,F_{2}^{8}\right)\times F_{2}^{8}\to F_{2}^{8}$ defined as

$$\begin{array}{c} f\left(t\right)=\frac{a_{1}t+a_{2}}{a_{3}t+a_{4}} \end{array}$$ (14)

In above expression, *f* is known as a linear fractional transformation (LFT) with *a*_1_, *a*_2_, *a*_3_ and $a_{4}\in F_{2}^{8}$ satisfying the condition *a*_1_ *a*_4_ − *a*_2_ *a*_3_ ≠ 0. The algebraic complexity and nonlinearity of LFT gives incentive to deploy this for the process of byte substitution. For our S-box, in particular, we choose *a*_1_ = 21, *a*_2_ = 8, *a*_3_ = 3 and *a*_4_ = 17. The images of this map produce our S-box as shown in Table 1. This highly nonlinear S-box attains the nonlinearity measure 112 that is quite similar to the recent state-of-the-art AES S-box but it utilizes a straightforward and comparatively simple approach as compared to the AES S-box. In the following section we use some highly significant analysis techniques to figure out the cryptographic strength of our S-box.

**Table 1:** LFT-based S-box.

215	93	171	23	234	76	201	236	175	59	141	214	99	162	108	74
167	97	3	36	235	95	52	1	60	242	55	161	63	110	225	241
145	153	245	254	73	17	118	90	173	21	178	176	94	122	136	114
72	177	43	58	56	11	184	149	120	127	185	37	243	157	69	10
189	92	77	0	196	222	4	223	181	168	78	186	207	195	148	190
50	66	26	70	238	112	132	248	221	46	253	2	102	188	247	170
194	187	45	53	213	86	62	24	200	115	111	68	212	40	140	130
104	163	98	82	119	31	7	154	255	155	81	15	85	219	42	64
20	80	129	211	88	160	156	218	123	109	204	107	19	205	12	216
106	84	30	169	228	44	249	135	124	229	159	232	67	133	126	101
137	100	38	144	143	116	29	134	244	180	224	217	33	113	6	210
203	158	22	166	79	138	105	164	183	240	65	191	209	197	27	251
150	227	239	51	12	61	54	165	48	237	233	147	41	193	252	198
206	230	25	87	89	28	47	16	151	96	35	172	57	152	199	139
220	117	246	71	208	34	121	13	83	32	128	103	39	146	75	167
14	179	131	91	226	182	231	174	18	49	142	5	8	9	192	202

## Performance analysis of S-box

In this section, we analyze the proposed S-box through some widely accepted parameters. The detailed analysis is presented in the following subsections. We compare the results with the famous S-boxes, as named earlier.

### Nonlinearity

The nonlinearity is a measure of the minimum distance of the reference function from the set of all the affine functions [2].

The average nonlinearity value for the proposed S-box is 112. A comparison of nonlinearity measure with some formerly prevailing S-boxes is shown in Table 2. Clearly, the nonlinearity of the proposed S-box matches the best attained figure.

**Table 2:** Comparison of performance indices of different S-boxes with LFT S-box.

S-box	Nonlinearity	SAC	BIC	DP	LP
**Proposed**	**112**	**0.510254**	**112**	**0.015625**	**0.0625**
AES	112	0.5058	112.0	0.0156	0.062
APA	112	0.4987	112.0	0.0156	0.062
Gray	112	0.5058	112.0	0.0156	0.062
Skipjack	105.7	0.4980	104.1	0.0468	0.109
Xyi	105	0.5048	103.7	0.0468	0.156
RP	99.5	0.5012	101.7	0.2810	0.132

### Strict avalanche criterion

This criterion is used to gauge the confusion creating capability of an S-box. A function $F:F_{2}^{n}\to F_{2}^{n}$ would be regarded to satisfy SAC if a single input-bit change assures change in 50% output-bits. The results presented in Table 2, shows that our S-box fulfils the requirements of SAC.

### Linear and differential approximation probabilities

This is a measure of the unevenness of an event, mathematically defined by:

$$\begin{array}{c} LP=\max_{\Gamma_{x},\Gamma_{y}\neq 0}|\frac{\#\{x|x.\Gamma_{x}=S\left(x\right).\Gamma_{y}\}}{2^{n}}-\frac{1}{2}|, \end{array}$$

where *x* represents all possible inputs to the S-box and Γ_*x*_ and Γ_*y*_ give the parity of the input and output bits respectively.

We further use the differential approximation probability, which determines the differential uniformity of an S-box. Its mathematical expression is given by;

$$\begin{array}{c} DP=[\frac{\#\{x\in X|S(x)⊕S(x⊕\Delta x)=\Delta y\}}{2^{n}}] \end{array}$$

Here Δ*x* and Δ*y* represent the input and out put differentials respectively. The smaller LP and DP measures guarantee the stronger S-box.

### Bit independence criterion

In bit independence criterion, input bits are altered exclusively, and then output bits are scrutinized for their independence. Bit independence has great worth in cryptographic structures. The goal of reaching the maximum complexity and perplexity in a system can be achieved through this property of increasing independence between the bits. In cryptographic systems, the increased independence between bits is an essential requirement as it makes harder to understand and forecast the design of the system.

The results of BIC are compared in Table 2. It is evident that, in BIC, our S-box has similarity with the Xyi S-box.

Based on the performance evaluation of the newly designed S-box, it is evident that the S-box shows extra-ordinary results and can be considered for further multimedia applications.

## Encryption method

The secret image is encrypted to keep the confidential information, the Fresnelet transform is employed based on the wavelet family, known as Haar. At first, the secret image Λ is propagated using the Fresnelet transform Δ_*τ*_ at the distance *d*_1_ = 1*m*, as follow:

$$\begin{array}{c} \Delta_{\tau }\left(\Lambda ,d_{1}\right)=\left(\begin{array}{cc} \Lambda_{\tau ,d_{1}}^{\left(ll\right)} & \Lambda_{\tau ,d_{1}}^{\left(hl\right)}\\ \Lambda_{\tau ,d_{1}}^{\left(lh\right)} & \Lambda_{\tau ,d_{1}}^{\left(hh\right)} \end{array}\right). \end{array}$$

In the second phase, a scrambled data *ψ* is generated from the components of decomposed image Λ using the *I*Δ_*τ*_ (inverse Fresnelet transform) at a distance, *d*_2_ = 10^−2^ *m*, as follow:

$$\psi =IF_{\tau }\left\{\left(\begin{array}{cc} \Lambda_{\tau ,d_{1}}^{\left(ll\right)} & \Lambda_{\tau ,d_{1}}^{\left(hl\right)}\\ \Lambda_{\tau ,d_{1}}^{\left(lh\right)} & \Lambda_{\tau ,d_{1}}^{\left(hh\right)} \end{array}\right),d_{2}\right\}.$$

The encrypted data from the secrete image are obtained in Fig 2 in form of complex properties of the Fresnelet transform. Moreover, complex data is separated into the imaginary part *ψ*_*im*_ and the real part *ψ*_*re*_ for getting unique pattern of non-linear pattern using S-box.

## Single unit dummy data

To manage the data the encrypted dummy data, we employ the inverse wavelet transform (WT) for reconstructing the two parts (real and imaginary) into single unit dummy data. The inverse wavelet with the Haar transform is employed.

The subband data *C*_0_ is the low-passed and the diagonal detail zeros valued data. The maximum intensity values of image $M\in R^{2}$ of horizontal detail and the vertical detail are used *α*_1_.*D*_*re*_ data and *α*_2_.*D*_*im*_ to derive the *α*_1_ and *α*_2_ as encryption normalization values. A scale parameter *α* is presented as a strength factor which regulates the participation of scrambled dummy data for getting single unit dummy data as follow:

$$\begin{array}{c} \alpha_{1}=\frac{\left|(M\left(D_{re}\right)|+|M\left(D_{re}\right)\right|}{\left|M(D_{re})\right|}, \end{array}$$

$$\begin{array}{c} \alpha_{2}=\frac{|M\left(D_{im}\right)|+|M\left(D_{im}\right)|}{\left|M(D_{im})\right|}, \end{array}$$

$$E=IWT\left(\begin{array}{cc} C_{0} & \alpha_{1}D_{re}\\ \alpha_{2}D_{im} & C_{0} \end{array}\right).$$

The above reconstruction process with the inverse wavelet transform (IWT) gives an information dummy data *E* after uniting the real and imaginary parts of *D* in reconstruction phase.

## S-Box encryption

**Fig 3:**
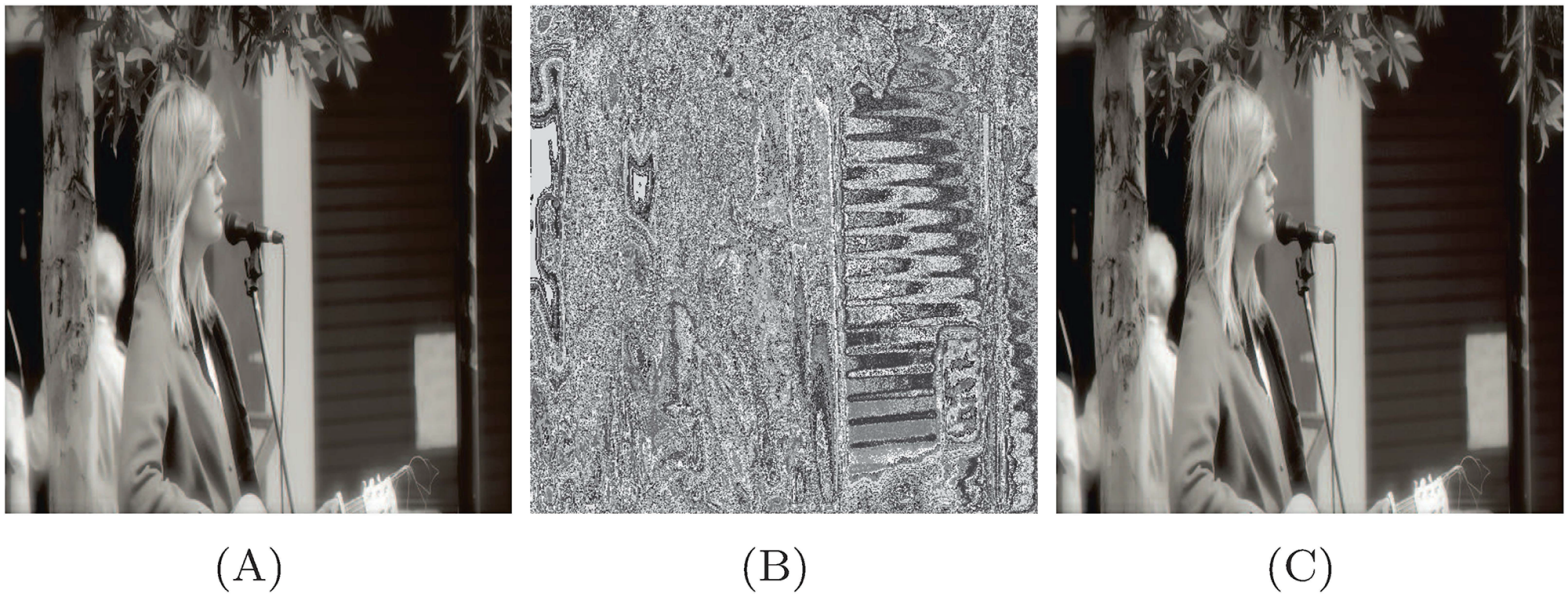
S-box image encryption and decryption of signature image (Signature image is taken from flicker image database: 293 https://www.flickr.com/photos/wyncliffe/14353455608/in/photostream/.) with various range of gray values (A) original image, (B) encrypted image, and (C) decrypted image, respectively.

**Fig 4:**
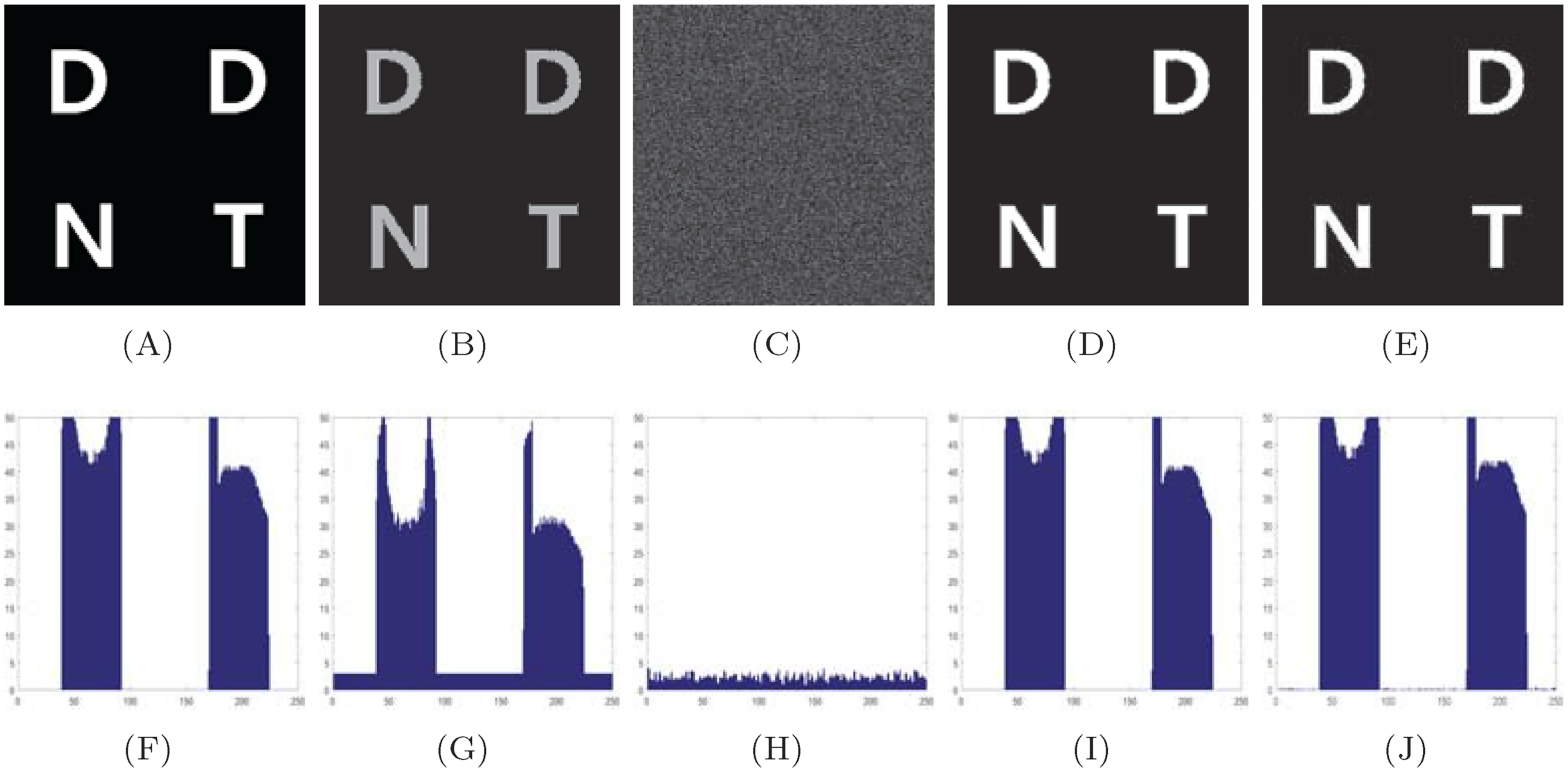
Visual and gradient estimation of S-box and propose method for highly contrast image (DDNT). (A) true image, (B) S-box encrypted image, (C) proposed method encrypted image, (D) decrypted image using inverse S-box, (E) decrypted image using inverse proposed method, (F)—(G) histogram gradient estimation of information data listed in first row from (A)-(E).

**Fig 5:**
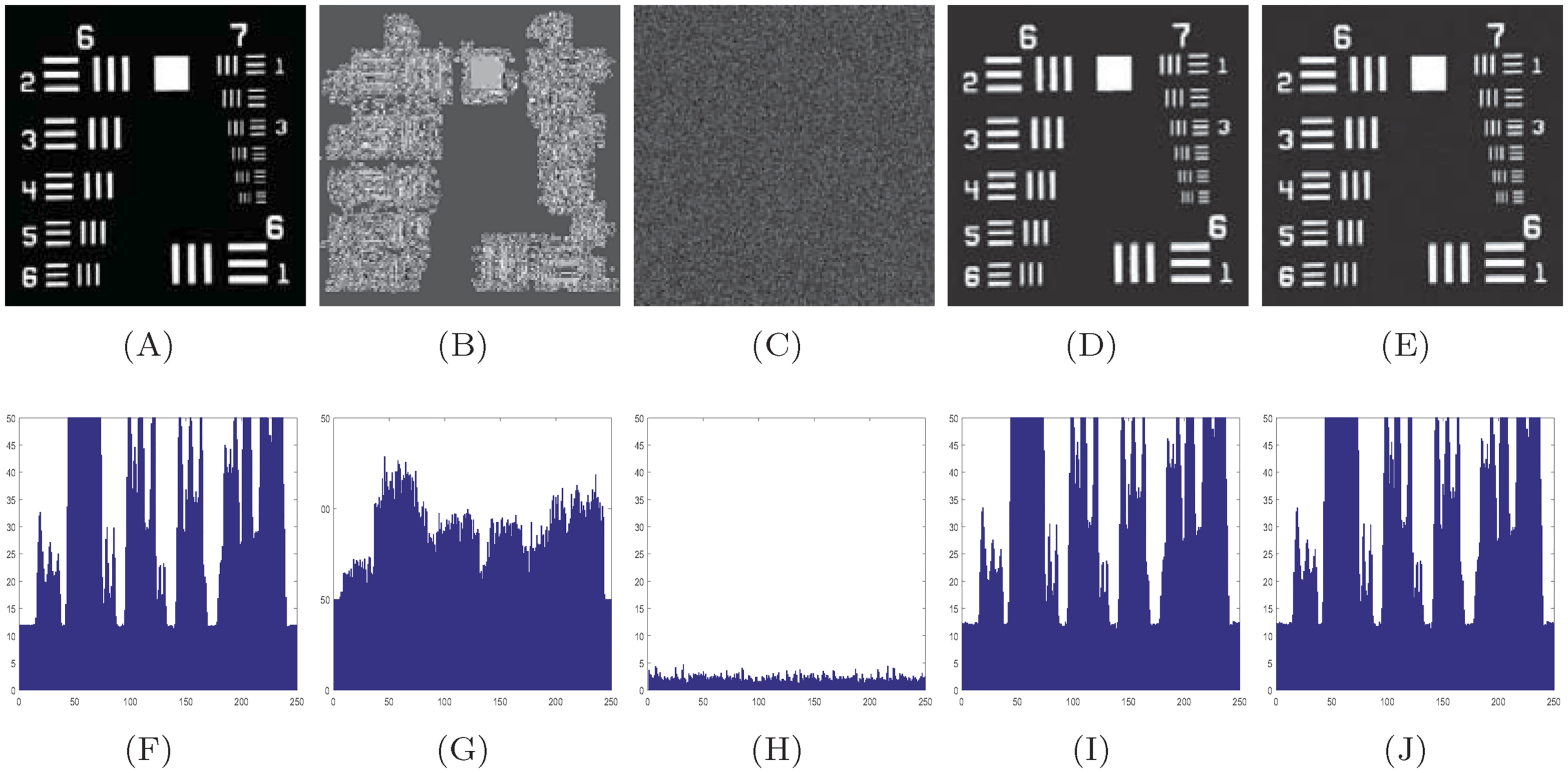
Visual and gradient estimation of S-box and proposed method for medium contrast image (USAF). (A) true image, (B) S-box encrypted image, (C) proposed method encrypted image, (D) decrypted image using inverse S-box, (E) decrypted image using inverse proposed method, (F)—(G) histogram gradient estimation of information data listed in first row from (A)-(E).

A typical 512 × 512 image of Signature, 256 × 256 image of DDNT, and 256 × 256 image of USAF are encrypted using the proposed method. We use the inverse of S-box to decrypt the ciphered image. Fig 3(A), 3(B) and 3(C) show the true, encrypted and the decrypted image of Signature. Figs 4 and 5 show the encrypted and the decrypted image of DDNT and USAF information data using S-box and the proposed method. One can see that our algorithm is capable to recover the true secret image with high accuracy as measured out in terms of coefficient correlation.

## The decryption process

The opposite of an encryption process is decryption process. The wavelet transform (WT) is considered to decompose the encrypted data image *E* into four subband data sets $E_{j-1}^{\left(ll\right)}$, $E_{j-1}^{\left(hl\right)}$, $E_{j-1}^{\left(lh\right)}$, and $E_{j-1}^{\left(hh\right)}$. The high frequency subband data $E_{j-1}^{\left(hl\right)}$ and $E_{j-1}^{\left(lh\right)}$ are carrier (communication) data sets that are preserved in the same position.

The final data are split by *α* so that the scrambled information data of same values are obtained. The retrieved imaginary and real parts of the scrambled data are rejoined in complex data pattern. Lastly the obtained complex scrambled data is refined by the inverse Fresnelet transforms applying the exact keys specification of the Fresnelet transform for the sake of obtaining the private information image on employing the inverse cipher S-box.

## Statistical analysis

In this section we gauge the security strength of the proposed algorithm by using some most significant statistical analysis, s described below.

- **Entropy**: Entropy analysis measures the randomness of a system. Table 3, represents the information entropy for different images (both original and the encrypted). It is obvious that the results favor the proposed methodology.
- **Contrast**: The contrast analysis is used to identify objects in an image. The numerical results for contrast of t images true and encrypted images are arranged in Table 3, which show that the proposed scheme is efficient.
- **Correlation**: The correlation coefficient, between the pixels at the same indices in both the plain and the encrypted image, measures the similarity between the pixels pattern of both images. Results arranged in Table 3 witness effectiveness of the proposed method.
- **Homogeneity**: The homogeneity analysis determines the closeness of the elements distribution in the gray level co-occurrence matrix (GLCM) to GLCM diagonal.
- **Differential analysis**: A desirable feature of an encryption algorithm is to show high sensitivity to single-bit change in the plain image. For this purpose two measures, NPCR and UACI, are commonly used. NPCR stands for the number of pixels change rate of image as a result of one pixel change in the plain image. However UACI means unified average intensity of differences between the plain and the encrypted images. In our case, the NPCR is over 99% and the UACI is over 33%. These results prove that our algorithm is highly sensitive and robust against the differential attacks and even single-bit difference in two plain image results in absolutely different encrypted images.

**Table 3:** Statistical analysis.

Image Test	Signature true	Signature encrypted	DDNT true	DDNT encrypted	USAF true	USAF encrypted
Entropy	7.4451	7.7512	0.0104	0.01609	0.4190	1.1379
Contrast	0.2100	8.6947	0.03602	0.0046	0.5509	-0.0014
Correlation	0.9444	0.1169	0.5928	0.0380	0.8330	0.8036
Homogeneity	0.9084	0.4524	0.9993	0.9996	0.9993	0.9284

## Simulation and evaluation

In the given process, we deal with a sampling interval size Δ = 10 *nm* of a hypothetical CCD plane, a wavelength λ = 632.8 *nm* (*nm* = *nanometer*), and distances *d*_1_ = 1*m* (*m* = *meter*) and *d*_2_ = 10^−4^ *m* [14]. These parameters are employed in the Fresnelet transform operations for the encryption as well as decryption stages and are considered as the key parameters. The obtained information images are predicted through the evaluation of correlation coefficients (CC) with the original information image data. The index of CC followed with in the range of 0 to 1. 0 value show almost no correlation between original and decrypted image while 1 reflect almost same out compare to original image.

To analyze the presented algorithm, our main focus on encryption analysis. The decrypted out put is almost clear with average value in terms of coefficient correlation value (0.9995). Notice from Table 3 that the given scheme provides good encryption of secret information data as compared to ordinary S-box encryption methods [2].

### Processing speed

It is an important feature of an efficient encryption algorithm to elapse lesser processing time. We tested our algorithm on MATLAB R-2016a, with *i*5 − 2520M CPU 2.50 GHZ and 8GB memory and the results are presented in Table 4. it is evident that our scheme is quite efficient.

**Table 4:** UACI, NPCR and Time.

	Signature	DDNT	USAF
UACI	33.7113	33.6721	33.1214
NPCR	99.3145	99.7813	99.7162
Time (Sec)	0.6128	0.6031	0.6114

### Classical attacks

By Kerckhoff’s principle, a sscure cryptosystem is one which can’t be broken even if everything is known, except for the secret key. Keeping this fact in view, classically, there are four main attacks [36], that are worth-studying.

- **Ciphertext only attack**: When a string of *ciphertext* is known to the attacker.
- **Known Plaintext attack**: When a string of *plaintext* along with the corresponding ciphertext is known to the attacker.
- **Chosen plaintext attack**: When attacker has temporary access to the encryption mechanism such that he can choose a *plaintext string* and obtain its ciphered version.
- **Chosen ciphertext attack**:
  When attacker has temporary access to the decryption mechanism such that he can choose a *ciphertext string* and can obtain its corresponding plaintext string.
  Among the above-mentioned attacks, *Chosen ciphertext attack* is of highest importance. If a cryptosystem can resist this attack, it would surely resist the other attacks as well [36, 40]. Obviously the proposed scheme is highly sensitive to the subband-distribution, the irreducible monic polynomial used to construct the background Galois field and the S-box parameters *a*_1_, *a*_2_, *a*_3_ and *a*_4_. Minor change in these will cause serious effects on the outcome and therefore our scheme is quite safe against the aforementioned attacks.

## Conclusion

To overcome the drawback of general S-box encryption in highly contrast data, this paper presents a new encryption approach with improved cipher security of the encrypted data. The proposed method is considered better approach for two significant reasons. First, the Fresnelet transform is used for encrypting the data of information image with various distance parameters as key. Second, the algebraic s-box provide highly nonlinearity in an encrypted data. Experimental studies validate that an encryption of digital image using the proposed scheme shown great superiority over the recently existing encryption methods. Although the proposed method in this study intents at the image encryption, however, its limitation does not restrict it to only this area and can be significantly implied in various fields of security information.

## Supporting information

Data Availability Statement: Signature image is taken from flicker image database: https://www.flickr.com/photos/wyncliffe/14353455608/in/photostream/. The person who associated a work with this deed has dedicated the work to the public domain by waiving all of his or her rights to the work worldwide under copyright law, including all related and neighboring rights, to the extent allowed by law. You can copy, modify, distribute and perform the work, even for commercial purposes, all without asking permission. Rest of the data used in the proposed method are available within the paper.
