# Correction: A novel encryption scheme for high-contrast image data in the Fresnelet domain

**DOI:** 10.1371/journal.pone.0196781

**Published:** 2018-04-26

**Authors:** Nargis Bibi, Shabieh Farwa, Nazeer Muhammad, Adnan Jahngir, Muhammad Usman

The affiliations for the first four authors are incorrect. Nargis Bibi is not affiliated with #1 but with #2, Department of Computer Science, Fatima Jinnah Women University, Rawalpindi, Pakistan. Shabieh Farwa, Nazeer Muhammad, and Adnan Jahngir are not affiliated with #2 but with #1, Department of Mathematics, COMSATS Institute of Information Technology, Wah Cantt., Pakistan.
