# Correction: A novel encryption scheme for high-contrast image data in the Fresnelet domain

**DOI:** 10.1371/journal.pone.0208305

**Published:** 2018-11-26

**Authors:** Nargis Bibi, Shabieh Farwa, Nazeer Muhammad, Adnan Jahngir, Muhammad Usman

There are incorrect values in Table 3 and Table 4. Please see the corrected Table 3 and Table 4 here.

**Table 3:** Statistical analysis.

Image Test	Signature true	Signature encrypted	DDNT true	DDNT encrypted	USAF true	USAF encrypted
Entropy	7.4067	7.4037	0.0104	0.01609	0.4190	1.1379
Contrast	0.1455	7.8170	0.03602	0.0046	0.5509	-0.0014
Correlation	0.9848	0.2777	0.5928	0.0380	0.8330	0.8036
Homogeneity	0.9429	0.5412	0.9993	0.9996	0.9993	0.9284

**Table 4:** UACI, NPCR and Time.

	Signature	DDNT	USAF
UACI	33.4421	33.6721	33.1214
NPCR	99.6107	99.7813	99.7162
Time (Sec)	0.6405	0.6031	0.6114
